# Convulsions from Dention

**Published:** 1861-11

**Authors:** 


Convulsions from Dentition.—The following given in "Hospital Practice" by Dr. Pepper, in the *Medical & Surg. Reporter*, is interesting and instructive, and affords matter for thought, both in the facts given, and in the suggestions made.

The subject of dentition and the pathological conditions that are dependent upon, or influenced by it, is one that is entirely too much neglected by dentists.

We hope the time is near at hand in which attention, study and investigation will be given by dentists to all things that exercise a controlling or modifying influence upon that department of the healing art with which they stand more immediately connected. Ed.

A child, 23 months old, up to eight weeks ago healthy, was taken with violent convulsions, which continued constantly for three weeks; the child is now paralyzed; no power of locomotion; loss of speech; eyesight unimpaired; is apparently imbecile; keeps its hands and its head in constant motion, and gazes about with a vacant stare. We have here the result of reflex irritation of dentition upon the brain, and, probably an effusion of blood or fibrin at the base of the brain. The prognosis is not unfavorable; the child is young; the deposit or effusion may be absorbed, and the brain be relieved of the pressure upon it. But in the treatment, care must be taken in regard to internal remedies. The remedial powers of nature, in cases of this character, are great, and some would, perhaps, trust to nature entirely; but the judicious administration of the proper remedy will, I think, aid nature in restoring the deranged functions. I should, therefore, advise the use of iodide of potash, in doses of half a grain, three times a day, believing this to be the remedy required. And further than this I should not go at present; it is too early in the case to give other medicines.

